# Molecular Mechanics Study of Flow and Surface Influence in Ligand–Protein Association

**DOI:** 10.3389/fmolb.2021.659687

**Published:** 2021-05-10

**Authors:** Shivansh Kaushik, Chia-en A. Chang

**Affiliations:** Department of Chemistry, University of Chemistry, Riverside, CA, United States

**Keywords:** molecular modeling, molecular recognition, drug design, GeomBD, ligand-receptor binding

## Abstract

Ligand–protein association is the first and critical step for many biological and chemical processes. This study investigated the molecular association processes under different environments. In biology, cells have different compartments where ligand–protein binding may occur on a membrane. In experiments involving ligand–protein binding, such as the surface plasmon resonance and continuous flow biosynthesis, a substrate flow and surface are required in experimental settings. As compared with a simple binding condition, which includes only the ligand, protein, and solvent, the association rate and processes may be affected by additional ligand transporting forces and other intermolecular interactions between the ligand and environmental objects. We evaluated these environmental factors by using a ligand xk263 binding to HIV protease (HIVp) with atomistic details. Using Brownian dynamics simulations, we modeled xk263 and HIVp association time and probability when a system has xk263 diffusion flux and a non-polar self-assembled monolayer surface. We also examined different protein orientations and accessible surfaces for xk263. To allow xk263 to access to the dimer interface of immobilized HIVp, we simulated the system by placing the protein 20Å above the surface because immobilizing HIVp on a surface prevented xk263 from contacting with the interface. The non-specific interactions increased the binding probability while the association time remained unchanged. When the xk263 diffusion flux increased, the effective xk263 concentration around HIVp, xk263–HIVp association time and binding probability decreased non-linearly regardless of interacting with the self-assembled monolayer surface or not. The work sheds light on the effects of the solvent flow and surface environment on ligand–protein associations and provides a perspective on experimental design.

## Introduction

Molecular association is the first critical step in all chemical and biological processes such as the immune response, signal transduction, drug-protein binding, and chemical catalysis (Ozbabacan et al., 2010; Dill and Bromberg, 2012; Baron and McCammon, 2013; Lin et al., 2020). Simulation techniques play a crucial role in investigating the environment that may affect ligand–protein binding, which provides a fundamental understanding of molecular recognition and reduces the cost and time in molecular design. Although diffusion-controlled association rate constants may be approximated analytically, most ligand–protein systems have slower association rates than the diffusion-limited rate because the association event involves multiple steps (Di Cera, 2017; Pang and Zhou, 2017). Conformational rearrangement of both molecules largely determines their binding kinetics, but the two molecules must have an initial encounter first. This first step may be greatly affected by the environment, which can result in different measured association-rate constants.

Although many ligand–protein bindings take place in a closed system without water flow such as in an experimental beaker or cell, ligand–protein association can also occur in a more dynamic environment. Different compartments within a cell also create various membrane environments when ligands and proteins associate and function (Zotter et al., 2017). For example, techniques using continuous flow biocatalysis have been developed recently to improve the efficiency of chemical synthesis, such as improved mixing, mass transfer, and automation (Planchestainer et al., 2017; Britton et al., 2018). Surface plasmon resonance (SPR), a powerful technique to measure molecular binding kinetics and thermodynamics, also utilizes flow chemistry and a continuous flow environment (Hinman et al., 2018; Prabowo et al., 2018; Ershov et al., 2020). However, how the flow may affect the ligand–protein association processes is unclear. Moreover, these methods need to immobilize one molecule on a surface that may have intermolecular interactions with the molecules to be tested. Therefore, the choice of surface and flow rate usually need to be optimized for various systems.

In addition to experimentally measured values such as association rate constants, molecular simulation can reveal atomistic details of molecular association to further interpret experimental results and understand binding mechanisms. Molecular encounter usually involves two molecules searching for each other in a vast space and for longer than a nanosecond search time. Brownian dynamics (BD) simulations have been used for computational assessment of the encounter processes for several decades (Northrup et al., 1984; Huber and McCammon, 2019). Many software packages are available for studying a variety of problems related to bimolecular association. For example, MacroDox, UHBD, BrownDye Simulation of Diffusional Association (SDA), BD_BOX, BROMOC, ReaDDy, Smoldyn, SEEKR, and BDpack are used to probe ligand–protein associations with a flexible or rigid biomolecular model (Madura et al., 1995; Northrup et al., 1999; Huber and McCammon, 2010; Długosz et al., 2011; Schöneberg and Noé, 2013; De Biase et al., 2015; Martinez et al., 2015; Saadat and Khomami, 2015; Andrews, 2017; Votapka et al., 2017). Our group has been developing the GeomBD program, which focuses on using BD to investigate inter-enzyme intermediate transfer and substrate association on surface environments in biosensor and nanoenzyme structures (Roberts and Chang, 2015, 2016). Because of the diverse bio-systems and binding environments, modifying an existing BD package to answer various questions is common practice.

Here we used the HIV protease (HIVp) and xk263 as a model system to study the effect of flow and surface on ligand–protein associations. HIVp belongs to the class of aspartyl proteases and is essential for maturation and assembly of infectious virions (Kohl et al., 1988). The protein cleaves the large polyprotein precursors to mature viral proteins (Huang et al., 2014), an essential function for viral replication, and is a major drug target for AIDS treatment. This is a well-studied system with rich experimental binding data for many inhibitors and FDA approved drugs (Ghosh et al., 2016). The flexible flaps of HIVp can serve as a gate: its opening and closing affects ligand binding. In proteins, an open/closed rate of a gate related to the diffusion of their binding partners determines a fast or slow gating for ligand binding (Szabo et al., 1982; McCammon, 2011). Because of the complex gating behavior of HIVp and its importance in therapeutics, earlier work applied BD to study drug–HIVp binding and also developed a specialized coarse-grained model for HIVp to model the large-scale motions of the flaps (Tozzini and McCammon, 2005; Chang et al., 2006; Kang et al., 2011; Li et al., 2012; Bernetti et al., 2019).

In this work, we used BD simulations to examine the xk263–HIVp association under the influence of ligand diffusion flux and a surface environment. The simulation applied rigid-body BD movements and considered atomistic details using pre-computed grids when computing intermolecular interactions and driving forces. The HIVp was immobilized on a surface or artificially placed 20 Å above the surface. We introduced a self-assembled monolayer (SAM) with a CH_3_ terminal group to model a hydrophobic surface environment (Cholko et al., 2019). We analyzed ligand association time and binding probability with different diffusion fluxes of xk263, surface and HIVp orientations. The non-polar SAM provided only weak intermolecular attractions with xk263, but the surface did not accelerate the xk263 encounter processes. The x-direction diffusion flux of xk263 significantly reduced weak intermolecular interactions and searching near the surface which shortened the association time but also significantly reduced the probability of successful binding. The concentration gradient of xk263 was affected by the xk263 diffusion flux as well. Our studies suggest that the flow may have noticeable effects on molecular encounter processes and provide insights into the differences in the ligand–protein association in diverse conditions, such as in static cells or a continuous flow biocatalysis environment.

## Methods

### Model System

**Figure 1:**
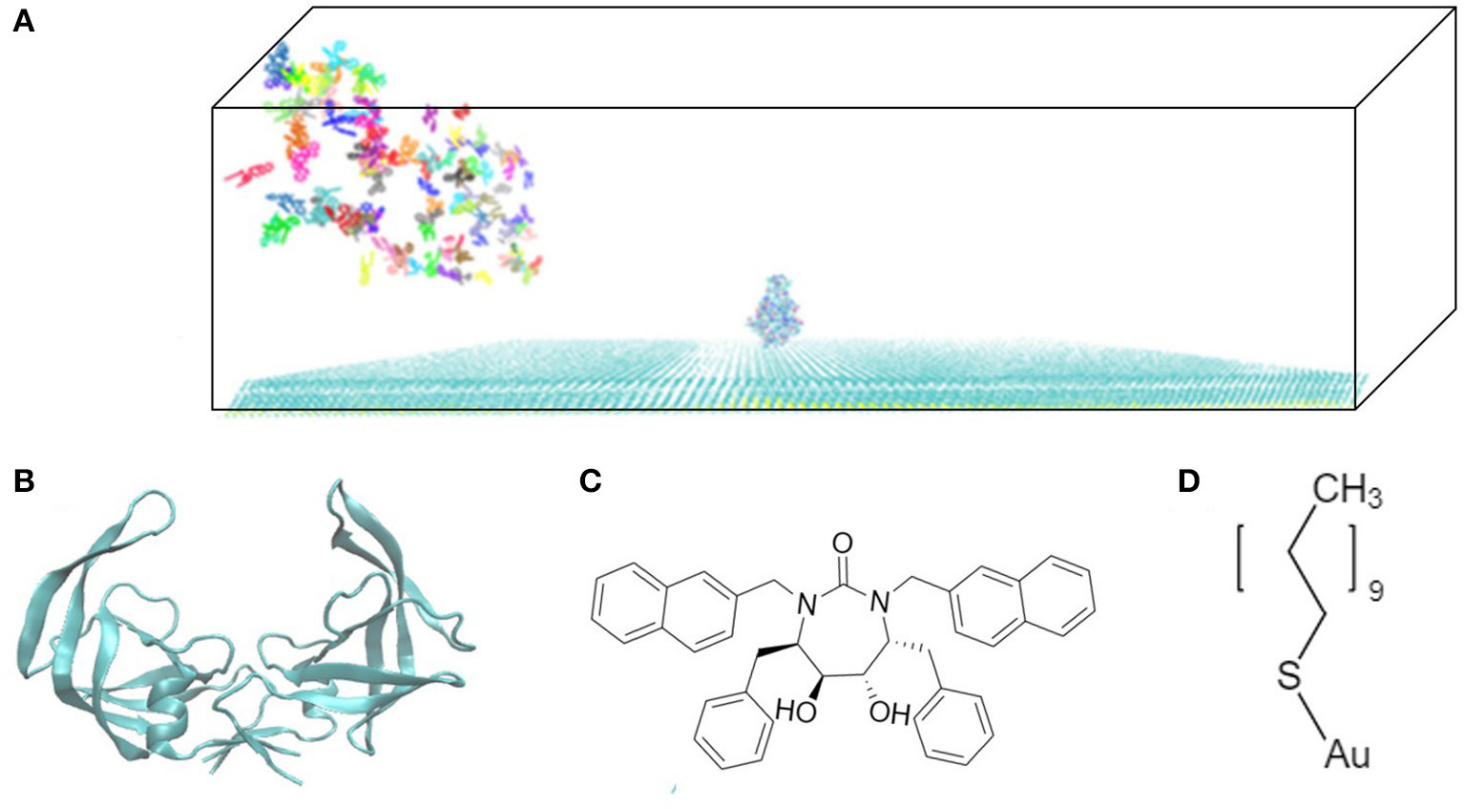
Model system. **(A)** HIVp is placed at the center of the CH_3_-SAM surface with xk263 ligands in a yz-plane when a simulation starts. **(B)** Structure of an open-form HIVp in cartoon representation. **(C)** xk263 ligand, and **(D)** undecanethiol molecule used to build the SAM surface. Notably, in each BD run, the molecular system has only one xk263 and one HIVp. To speed up the calculations, multiple replicas of xk263 are simulated simultaneously, but the replicas do not interact with each other.

**Figure 2:**
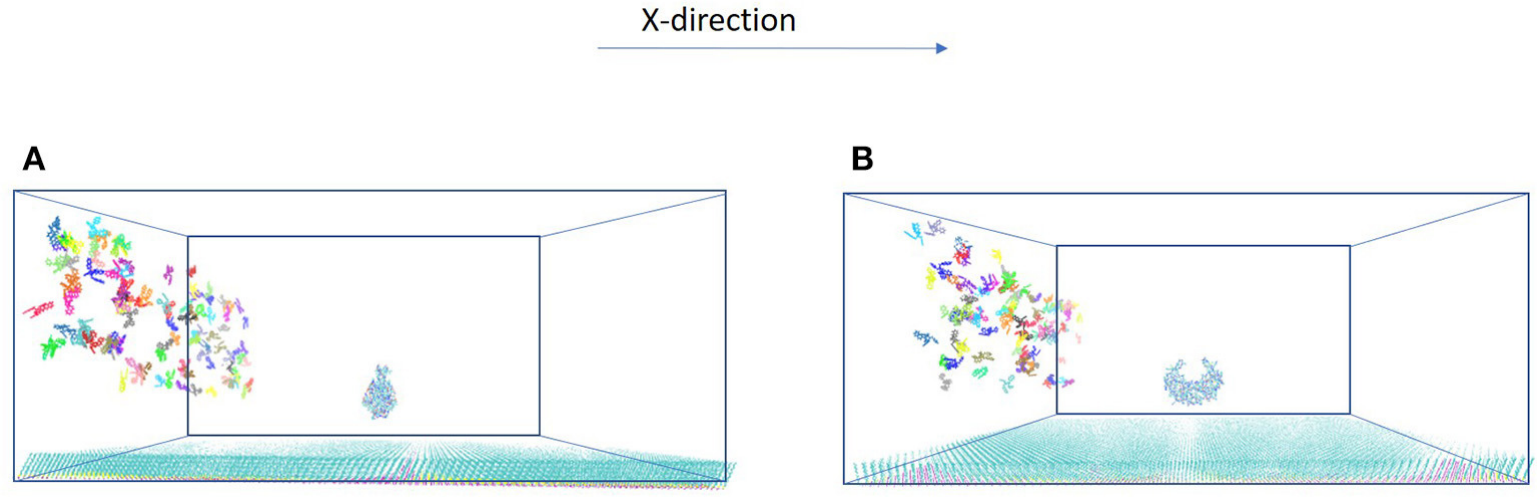
Two orientations of HIVp. The protein is placed 20 Å above the SAM **(A)** parallel to the yz-plane and **(B)** perpendicular to the yz-plane.

The model system is a rectangular prism (400 × 400 × 220 Å^3^), closed at the top, and consisting of HIVp, xk263, and a SAM surface (Figure 1A). The crystal structure of HIVp and the xk263 inhibitor were obtained from the Protein Data Bank (codes 1HHP and 1HVR, respectively) (Spinelli et al., 1991; Lam et al., 1994). The HIVp flaps, two polypeptides that cover the active site, are in the open position. The semi-open form crystal structure of HIVp was simulated to derive the open form (Supplementary Methods) (Huang et al., 2017). HIVp was kept fixed in the model (Figure 1B). The SAM surface with HIVp at the center consisted of undecanethiol chains on a gold sheet of 1 atom thickness (Supplementary Methods) (Cholko et al., 2019). The size of the SAM was 400 × 400 Å^2^ having a hexagonal packing pattern with a packing density in the order of 10^14^ cm^−2^, which gives an average chain separation of 4.98 Å. The system had a periodicity in the y-direction and termination boundary in the +x-direction as the ligand flows in the +x-direction. HIVp had two orientations (Figure 2).

### Simulation Details

The in-house modified GeomBD2 program was used for all BD runs (Roberts and Chang, 2016). The program first creates three grid files: the exclusion volume, screened Coulumbic potential, and 12-6 Lennard-Jones potential for HIV and SAM. Intermolecular interactions between ligand xk263 and HIVp or SAM are grid-based calculations, and the forces are estimated numerically (Roberts and Chang, 2016). To speed up the calculations, these precalculated grids are extended up to 40 and 15 Å around HIVp or SAM for screened Coulumbic and 12-6 LJ potentials, respectively. The force field parameters were obtained from Amber-ff14SB for HIVp and CH_3_-SAM. The partial charges of xk263 and SAM were computed by using the AM1-BCC charge method with the antechamber program (Cholko et al., 2019). The motion of the ligand is a Brownian motion, governed by an overdamped Langevin equation, in implicit water (Northrup et al., 1984).

(1) $$\begin{array}{r} r_{i}\left(t+\Delta t\right)=r_{i}\left(t\right)-\frac{D_{i}}{k_{B}T}\frac{\partial E}{\partial r_{i}}\Delta t+\sqrt{2D_{i}\Delta t}R \end{array}$$

where D_i_ is the translational or rotational diffusion coefficient, k_B_ is Boltzmann's constant, T is temperature, δE/δr_i_ is the potential gradient computed numerically based on the potential grids, Δt is the time step, and R is the stationary Gaussian random number with a zero mean. To obtain the flow for the ligand, the overdamped Langevin equation is modified by adding *N*, a small fractional number, in the displacement of the x-direction.

(2) $$\begin{array}{r} x_{i}\left(t+\Delta t\right)=x_{i}\left(t\right)-\frac{D_{i}}{k_{B}T}\frac{\partial E}{\partial x_{i}}\Delta t+\sqrt{2D_{i}\Delta t}R+N \end{array}$$

For each molecular system, we terminated the simulation when the runs generated at least 600 xk263-HIVp associates and the computed average association time was within the standard deviation of <3%. A successful xk263–HIVp association is defined as xk263 reaches within 11.5 Å ASP25, a residue in the active site of HIVp, and the association time is recorded. Notably, after generating 600 successful bindings, the computed average associating time for each system is within 3%.

**Figure 3:**
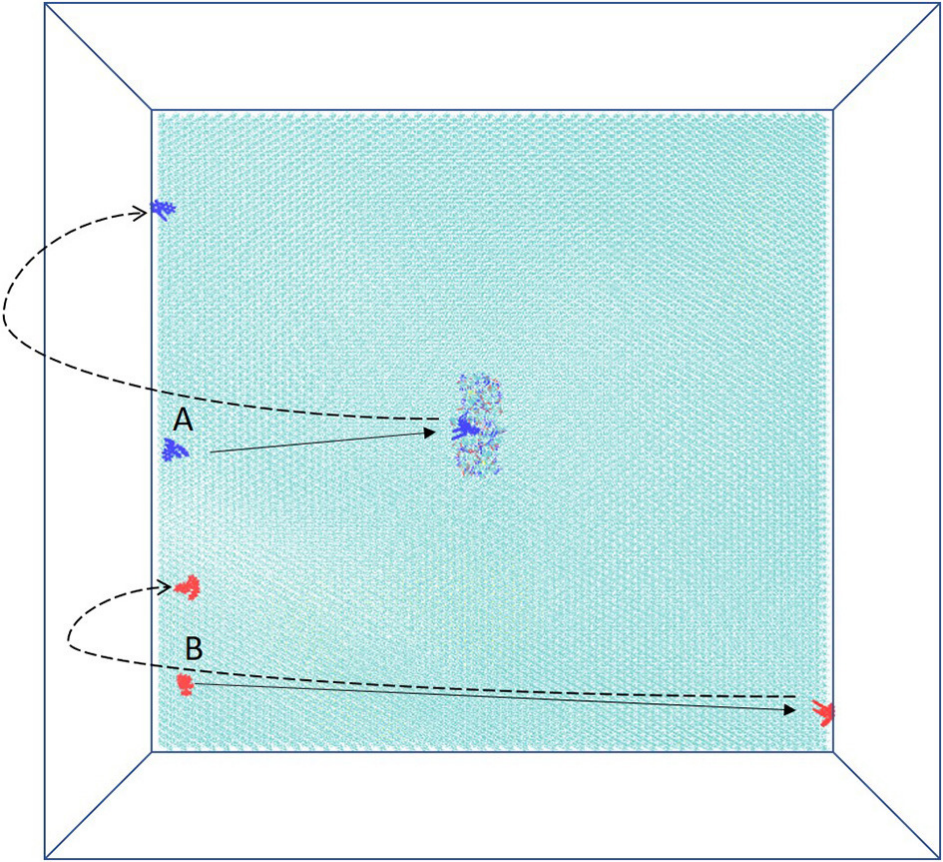
Schematic top view of the simulation box. HIVp is placed on CH_3_-SAM, and two initial starting positions of xk263, A and B, are on the yz-plane. The simulation is terminated after xk263 binding to HIVp, and another new replica will start. If xk263 reaches the +x-direction wall, the simulation is also terminated, and a new replica will start in a different position on the yz-plane.

In the system, xk263 starts diffusing from the yz-plane at the -x-direction boundary (Figure 1A). A run is ended when xk263 reaches the binding pocket or the +x-direction boundary, and another new run will be started by randomly placing xk263 in a new position on the yz-plane (Figure 3). The clock random number seed is used, so no two runs can be identical. In addition, we can save trajectories of our BD runs to visualize ligand diffusion and binding/unbinding events.

### Calculations for System Analysis

For the calculations of diffusion flux, diffusion coefficients, CH_3_-SAM interaction, and xk263 distribution, we used the trajectories with no termination at the +x-direction boundary. So, the trajectories used for the calculations are continuous and 0.3-μs long for each case.

#### xk263 Diffusion Flux

The diffusion flux is the rate of molecules transferred across the plane per unit time per unit area, representing the flow of xk263. It was calculated by using the continuous BD trajectories and reported in units of molecules/s.m^2^. The plane is an imaginary plane at the +x- directional boundary perpendicular to the direction of flow. The calculated flux densities according to the N values (in Equation 2) are reported (Supplementary Table 1).

#### Diffusion Coefficient

The diffusion coefficient was calculated by Einstein's relation, < *r*^2^> = 2nDt, where r is the displacement in time t, n is the dimensionality, and D is the diffusion coefficient.

#### CH_3_-SAM–xk263 Interaction Energy

To calculate the interaction energetics of xk263 with CH_3_-SAM, we used the selected portion of continuous trajectories with xk263 diffusions within 25 Å above the CH_3_-SAM. The interaction energy includes 12-6 Lennard-Jones potential and screened Coulombic potential. The cutoffs for vdW and electrostatic potential were 12 Å and 40 Å, respectively.

The calculation of 12-6 Lennard-Jones potential is as follows:

(3) $$\begin{array}{r} E_{vdw}=4\in \left(\frac{\sigma^{12}}{r_{ij}^{12}}-\frac{\sigma^{6}}{r_{ij}^{6}}\right) \end{array}$$

where *r*_*ij*_ is the distance between atoms *i* and *j*, ϵ is the pairwise well-depth parameter and σ is the distance at which the atomic radii meet and where the potential changes sign.

(4) $$\begin{array}{r} \sigma =\frac{\sigma_{i}{+\sigma }_{j}}{2} \end{array}$$

(5) $$\begin{array}{r} \epsilon =\sqrt{\epsilon_{i}\epsilon_{j}} \end{array}$$

The calculation of screened Coulombic potential is as follows:

(6) $$\begin{array}{r} E_{SC}=\frac{k_{e}q_{i}q_{j}e^{-kr_{ij}}}{r_{ij}\epsilon } \end{array}$$

where r_ij_ is the distance between two point-charges i and j from the ligand and receptor, respectively, q_i_ and q_j_ are the partial electron charges of the ligand point charges i and receptor point charges j, k is the screening parameter, k_e_ is the Coulomb constant, kb is the Boltzmann constant and ϵ is the solution dielectric constant.

#### xk263 Distribution

The distribution of xk263 in the system was calculated by using continuous BD trajectories. The volume of the system was divided into 11 slices of 20 Å height each. The total number of appearances of xk263 replicas was computed for each slice in 0.3 μs. The calculation of *g(z)*, distribution, is according to Equation 8

(7) $$\begin{array}{r} g\left(z\right)=\frac{N}{M}=\frac{N}{n_{R}\times \frac{v}{V}\times f} \end{array}$$

where N is the total number of appearances of xk263 replicas in a slice within a certain number of frames, M is the expected total number of appearances approximated from a random diffusional motion, n_R_ is the number of xk263 replicas in the system, v is the volume of the slice, V is the total volume of the system, and f is the number of frames to be analyzed.

## Results and Discussion

**Figure 4:**
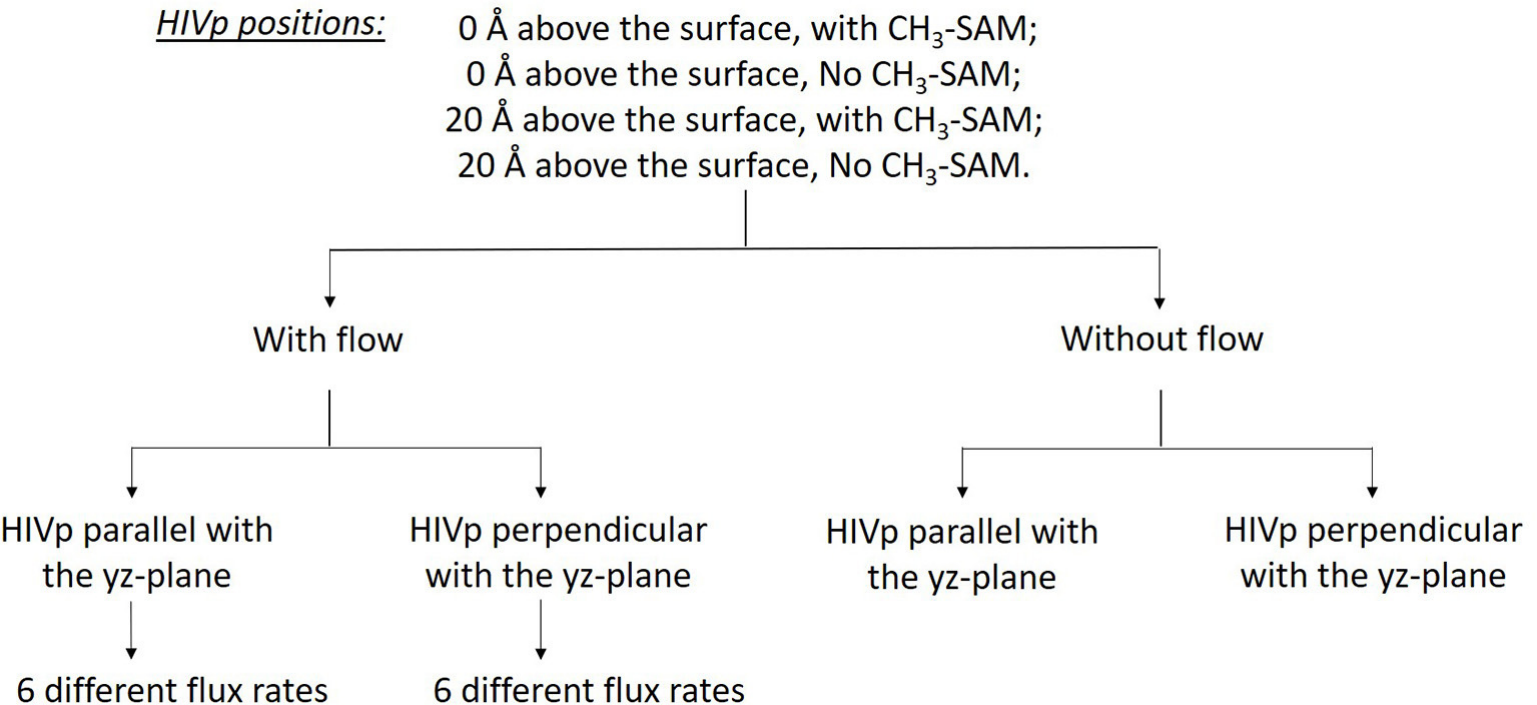
Sixty different systems used in this study. The two HIVp orientations are shown in Figure 2.

Understanding ligand–receptor associations in various environments is of vital importance in drug binding in cells or in different experiments. In this study, we analyzed the effects of the SAM surface, the diffusion flux of ligand xk263 and the position of HIVp from the surface and in comparison with results with a static environment (Table 1). HIVp has flexible flaps, which are mostly in closed conformations (Katoh et al., 2003; Kang et al., 2011). To bind with a ligand, the flaps may open spontaneously or are induced to open by the ligand. Because this study focuses on the initial molecular encounter processes, we chose a flap open conformation and used rigid-body BD simulations to model the ligand association. In addition, the orientation of HIVp with respect to the +x direction diffusion flux of xk263 can affect ligand association. Therefore, we examined two HIVp orientations, perpendicular and parallel with the ligand flux (Figure 2). We modeled 60 different systems (Figure 4). We chose the values of xk263 diffusion flux that gradually show the changes of the modeled values with the flux.

**Table 1:** Summary of molecular systems used in this study.

**Details of model system**	
HIVp orientations	2
Periodic wall	y
Flow direction	x
Top	Closed in z-direction
Ligand concentration	110 μM
Ligand initial position	As a yz-plane
Surface	with and without CH_3_-SAM
Protein height	0 and 20 Å above the surface
Box dimension	400 × 400 × 220 Å^3^
Termination	Reaching +x-direction wall or within 11.5 Å of ASP 25′

### Environmental Factors Involved in Ligand Diffusion and Distribution

#### Intermolecular Interactions Between xk263 and CH_3_-SAM

We first examined the interactions between xk263 and CH_3_-SAM when the ligand diffusion flux varies. Table 2 shows weak van der Waals (vdW) interactions between xk263 and CH_3_-SAM regardless of the flux, which resulted in no significant adsorption of xk263 on SAM (Figure 6A). Because of the hydrophobic nature of CH_3_-SAM and xk263, it is not surprising that the electrostatic interaction was close to zero.

**Table 2:** Interaction energies between xk263 and CH_3_-SAM in 7 different xk263 diffusion fluxes.

**xk263 diffusion flux (× 10^**22**^/s.m^**2**^)**	**E_**elec**_ (kcal/mol)**	**E_**vdW**_ (kcal/mol)**
0	−0.045 ± 0.14	−1.126 ± 0.89
0.45	−0.056 ± 0.12	−1.098 ± 0.65
1.09	−0.010 ± 0.11	−1.547 ± 1.14
3.43	0.015 ± 0.14	−1.194 ± 0.95
5.62	0.003 ± 0.11	−1.488 ± 0.96
11.29	0.006 ± 0.10	−1.273 ± 1.13
17.20	0.005 ± 0.13	−1.069 ± 0.83

**Figure 5:**
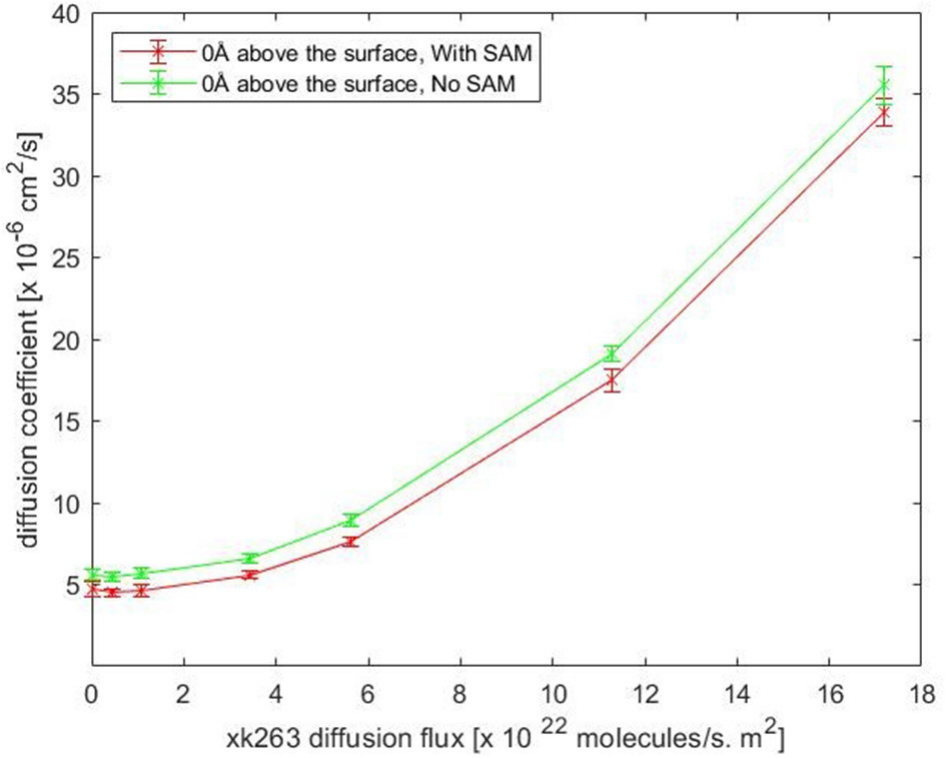
Plot of translational diffusion coefficient of xk263. Diffusion coefficient increases with xk263 diffusion flux. (see Supplementary Table 1 for numerical data).

Notably, our BD movement yielded the translational diffusion coefficient of xk263, D_lig_ = 5.58 ± 0.33 × 10^−6^ cm^2^/s when a system had no SAM and no ligand diffusion flux. The modeled ligand diffusion coefficient is in good agreement with analytical values, D_analytical_ = 4.91 × 10^−6^ cm^2^/s, which also validates our simulation setting and BD algorithm (Supplementary Methods). Although the intermolecular vdW was weak, the attractions still affected the diffusion of xk263 molecules, and the molecule diffused a little slower in the presence of SAM with or without xk263 diffusion flux (Figure 5, Supplementary Table 1). Because the flow rate is inversely proportional to the viscosity (Pfitzner, 1976), the diffusion coefficient of xk263 increased with increasing xk263 diffusion flux, as anticipated. However, because the water flow rate was not equal to the xk263 diffusion flux, we did not see a simple linear relationship between the two values.

#### Distribution of xk263 in the Simulation Tube

**Figure 6:**
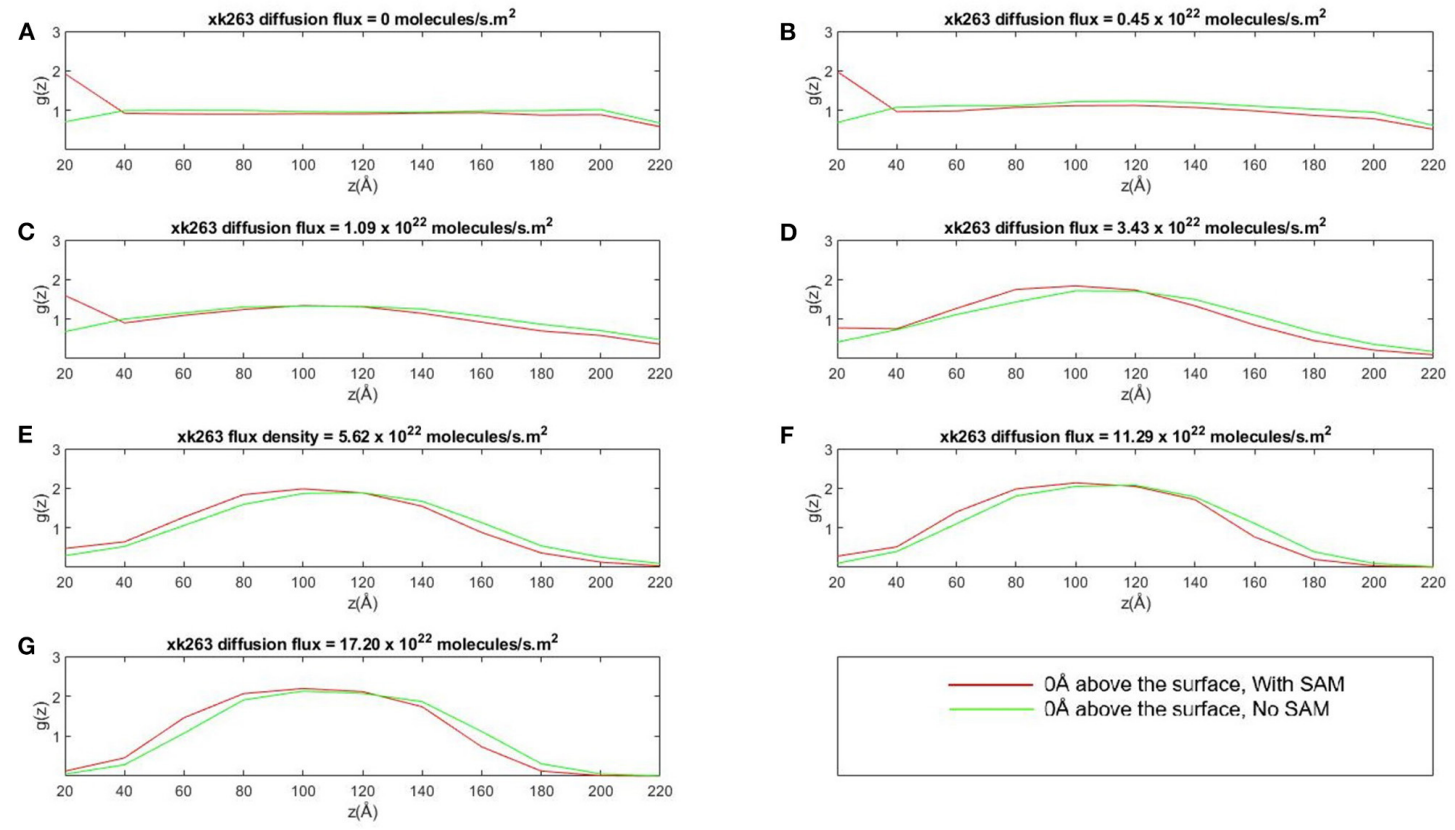
Plot of concentration distribution of xk263, g(z), as a function of the distance above the CH_3_-SAM, z, in seven different diffusion fluxes **(A–G)**. The space was partitioned into equally sized slices with a height of 20 Å. g(z) is the ratio of number of observed xk263 and the number of expected xk263 approximated from a random diffusional motion.

We further investigated whether the environment may affect the concentration gradient of xk263 along the z-direction when the molecule diffuses in the square tube. The system was partitioned into slices of 20 Å each along z-direction, and Figure 6 shows the distribution of xk263 as a function of their distance above the surface. The distribution of xk263 for a slice, g(z), is the ratio of number of observed xk263 and number of expected xk263 approximated from a random diffusional motion. The systems include HIVp with one orientation shown in Figure 2A. When there was no SAM and the xk263 diffusion flux was small (green line in Figure 6A), the distribution of xk263 was the same throughout the z-direction (distribution ratio = ~1). In contrast, when CH_3_-SAM was present (Figures 6A–C), xk263 was double that in the first slide (within 20 Å of the SAM) when diffusion flux of xk263 was <1.09 × 10^22^ molecules/s m^2^. The results suggest that even if the intermolecular attraction between xk263 and CH_3_-SAM is weak, the concentration can be increased by 2-fold, which helps to increase the probability of a molecular encounter. Studies for various systems also showed that local molecular concentration can be effected by a surface; for example, surface catalyzed biomolecular reaction using nanoreactors, nucleotidase co-localization, and absorption of biomacromolecules on hydrogels (Roa et al., 2017; Pérez-Mas et al., 2018; Rahmaninejad et al., 2020).

The SAM retains xk263 near the surface only when the diffusion flux of the ligand was small. When the flux increases, the local concentration of xk263 near the surface decreased, and xk263 had the highest concentration near the middle of the z-direction (see ~100 Å in Figures 6D–G). The distribution of concentration gradient along the z-direction was the same with or without SAM, which suggests that the weak intermolecular interactions can be altered easily when xk263 has diffusion flux in the x-direction. Our results are consistent with experiments showing the quadratic curve of concentration gradients with flow (Chen et al., 2017). With larger diffusion flux, a significantly smaller amount of xk263 diffused near the surface where HIVp locates, and the reduced distribution also contributed toward probability of small xk263–HIVp association, discussed later.

### HIVp–xk263 Association in Different Environments

#### HIVp Immobilized on the Surface

We modeled the average xk263–HIVp association time and the binding percentage when HIVp was 0 Å above the surface with SAM (Tables 3, 4 with SAM) or without SAM. Theoretically, if xk263 employs only 3-dimensional diffusion within a sphere, the average association time to bind to HIVp located in the center of the sphere is 2648.4 ns when the system has no external flux (Supplementary Methods) (Adam and Delbrück, 1968). The association time modeled by our simulation is 1491.55 ns is close to the theoretical value. The faster binding time than theory is due to use of a box, and xk263 did not need to search the whole sphere to associate with HIVp. Notably, existing association rate theories describe system without diffusion flux (Berg and Purcell, 1977; Szabo et al., 1980; Shoup and Szabo, 1982) and further theoretical work in molecular association with buffer flowing is needed. Although the interactions between xk263 and CH_3_-SAM were small, to examine the environment effects, we turned off the intermolecular vdW and electrostatic interactions, so the system was in a confined space without intermolecular attractions or repulsion (without SAM). The x-direction diffusion flux of xk263 gradually increased from zero to 1.7 × 10^23^ molecules/s m^2^, and the average xk263–HIVp association time decreased non-linearly. The correlation was observed in a previous study, showing that the association time for human immunoglobulin G (IgG) and *Staphylococcus aureus* protein A (SpA) decreased with increasing flow rate (Ogi et al., 2008). The average association time did not differ without or with SAM in the presence of diffusion flux (Table 3). This is not surprising because the CH_3_-SAM provides only weak attractions to xk263, and the surface neither increased the local concentration of xk263 to assist ligand association nor retained the ligand from binding to HIVp (Figure 6) (Cholko et al., 2020). However, if the attraction between a ligand and surface is large, it may affect experimental results. As a result, existing experimental studies considered the choice of the surface for the sensor or SPR to optimize experimental sensitivity and accuracy. For example, phospholipid/alkane bilayers might be a better option for molecular binding in biomolecular systems using SPR (Plant et al., 1995). These phospholipid derivatized surfaces may bring non-specific interactions for xk263 which is absent in our current model, which can result in slightly longer association time.

**Table 3:** Average association time (t_avg_) with different xk263 diffusion fluxes.

	**HIVp perpendicular to the yz–plane, t**_****avg****_ **(ns)**				**HIVp parallel to the yz-plane, t**_****avg****_ **(ns)**			
**xk263 diffusion flux (× 10^**22**^ /s.m^**2**^)**	**0Å above the surface, With SAM**	**0Å above the surface, No SAM**	**20Å above the surface, With SAM**	**20Å above the surface, No SAM**	**0Å above the surface, With SAM**	**0Å above the surface, No SAM**	**20Å above the surface, With SAM**	**20Å above the surface, No SAM**
0	1491.55	1064.41	1213.45	901.99	1405.77	1084.49	1124.5	905.21
0.45	126.50	122.00	108.70	110.17	145.54	124.90	128.30	111.68
1.09	55.56	51.93	43.61	45.28	53.71	50.05	47.98	44.65
3.43	18.73	18.83	15.042	15.57	17.77	17.83	16.23	15.53
5.62	11.08	10.48	10.31	9.47	10.78	11.01	9.75	9.55
11.29	5.62	5.52	5.52	4.80	5.53	5.38	4.89	4.89
17.20	3.69	3.59	3.33	3.25	3.58	3.69	3.27	3.26

**Table 4:** Inverse of average association time per molarity and percentage of successful binding with different xk263 diffusion fluxes.

**xk263 diffusion flux (×10^**22**^ /s.m^**2**^)**	**0Å above the surface, With SAM**		**0Å above the surface, No SAM**		**20Å above the surface, With SAM**		**20Å above the surface, No SAM**	
	**1/t_**avg**_ perM (/nsM)**	**Association percentage**	**1/t_**avg**_ per M (/nsM)**	**Association percentage**	**1/t_**avg**_ per M (/nsM)**	**Association percentage**	**1/t_**avg**_ per M (/nsM)**	**Association percentage**
**HIVp perpendicular to the yz-plane**								
0	6.09	18.59	8.54	18.14	7.49	18.67	10.08	19.18
0.45	71.86	3.02	74.52	3.82	83.63	3.53	82.52	4.24
1.09	163.63	1.22	175.07	1.73	208.45	1.67	200.75	2.16
3.43	485.44	0.52	482.66	0.64	604.35	1.00	583.76	0.89
5.62	820.41	0.35	867.45	0.33	881.41	0.90	959.31	0.65
11.29	1618.17	0.18	1646.61	0.14	1646.43	0.70	1892.99	0.43
17.20	2461.66	0.11	2532.29	0.07	2732.55	0.46	2793.33	0.34
**HIVp parallel to the yz-plane**								
0	6.47	17.15	8.38	17.85	8.08	18.99	10.04	20.61
0.45	62.46	3.25	72.78	3.88	70.86	3.43	81.40	4.22
1.09	169.25	1.21	181.64	1.71	189.48	1.86	203.61	1.92
3.43	511.39	0.38	509.78	0.48	559.99	0.71	585.19	0.75
5.62	842.87	0.22	825.62	0.21	931.88	0.40	952.29	0.42
11.29	1645.34	0.05	1690.36	0.05	1858.74	0.14	1858.13	0.15
17.20	2538.08	0.02	2461.19	0.02	2781.88	0.06	2788.36	0.07

We report the inverse of association time per molarity and the association percentage (Table 4). When xk263 was freely diffused in the tube without diffusion flux, the association rate approximated using the average association time, 1/1491.55 ns per M, yielded k_on_ = 7.8 × 10^9^ 1/Ms. Using the diffusion coefficient from the BD run (Supplementary Table 2), our simulated k_on_ was approximately a half of the analytical value, k_on_ = 4πRD = 1.2 × 10^10^ 1/Ms because the analytical formula does not consider molecular geometry in association. Our modeled k_on_ was also slightly slower than that measured using SPR for another highly similar cyclic urea compound, 2.5 × 10^10^ 1/Ms (Markgren et al., 2002). The first phase in SPR utilizes buffer flowing and detects binding signals, which require successful collision to have the correct ligand and HIVp orientation and enough energy. The flow may influence successful collision, especially on biomolecular systems which usually have complex molecular geometry. Of note, the inverse association time increased linearly with xk263 diffusion flux (Supplementary Figure 1), which suggests that the association rate may also increase linearly with the flow. However, our modeled diffusion coefficients (Supplementary Table 2) show an exponential increase with the xk263 diffusion flux (Figure 5). When particles have flux moving along the +x direction, the ligand did not freely diffuse in all directions, and the equation for the diffusion-controlled association system, k_on_ = 4πRD, was no longer suitable to approximate the association rate constants. Therefore, we cannot directly estimate k_on_ from the increased diffusion coefficient.

The additional +x direction drift velocity also reduced the initial encounter probability. When the flux was >3.43 × 10^22^ molecules/s.m^2^, xk263 passed the tube without sufficient time to freely diffuse in the y and z directions, which resulted in reduced search space and tremendously decreased association percentage. Large diffusion flux also yielded fewer xk263 molecules staying near both the SAM and HIVp surface. Notably, xk263 may diffuse on the surface of HIVp before binding to the pocket. The xk263 diffusion flux in the +x direction perturbed the interactions between xk263 and HIVp, and the weakened intermolecular attractions also led to decreased association percentage.

**Figure 7:**
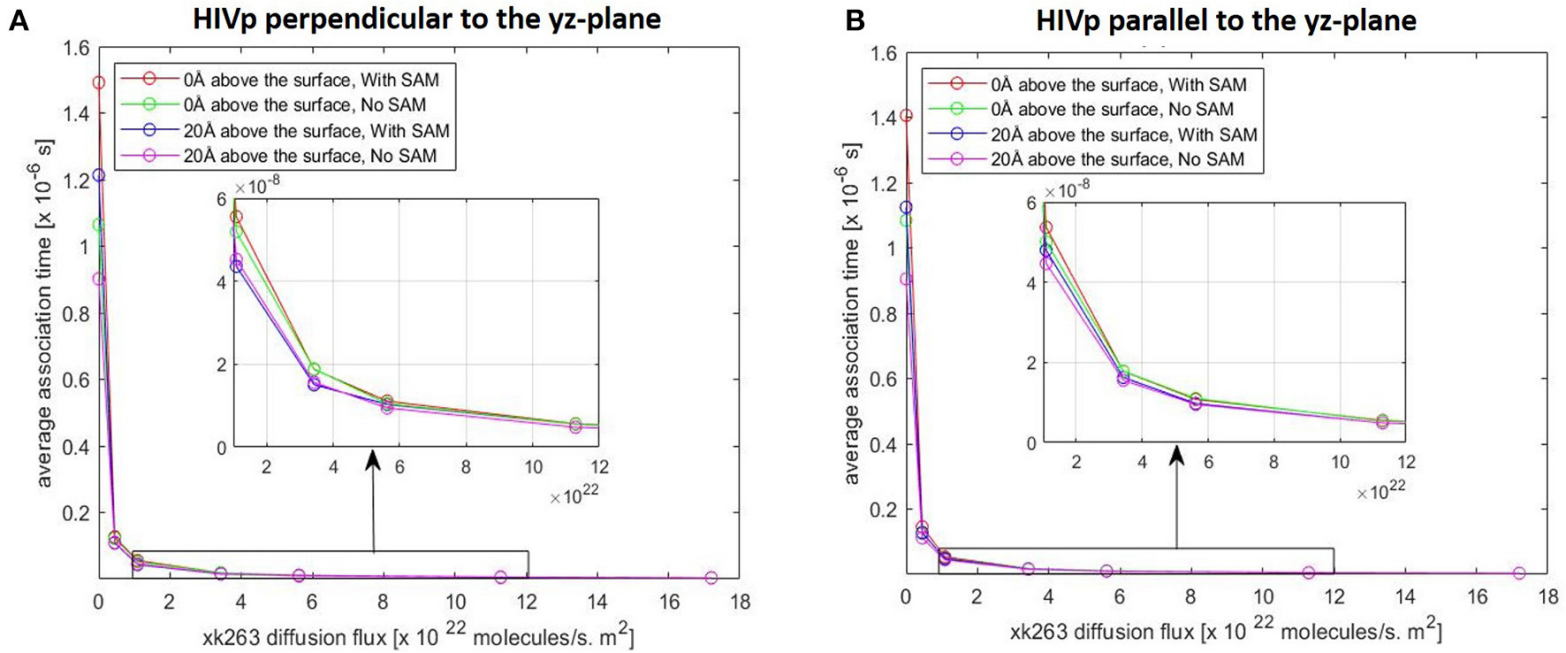
Plot of average association time vs. xk263 diffusion flux. HIVp is placed **(A)** perpendicular to the yz-plane and **(B)** parallel to the yz-plane; Similar data imply no effect of HIVp orientation on the association time. In each plot, coinciding lines (red, green, blue, and magenta) show no effect of hydrophobic CH_3_-SAM or height of the receptor from the surface.

**Figure 8:**
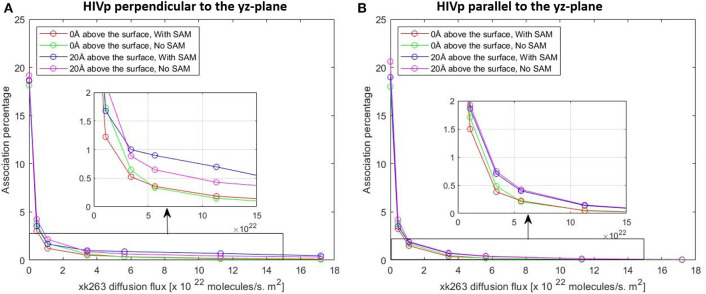
Plot of association percentage vs. xk263 diffusion flux under two different HIVP's orientation **(A,B)**. Association percentage is defined as the fraction of successful binding from the total number of trajectories in the system.

In typical experimental settings, an immobilized protein can have multiple orientations. Therefore, we chose two representative orientations—the binding pocket of HIVp perpendicular or parallel to the flux of xk263—to examine whether the orientation against the flow affects ligand binding. Tables 3, 4, Figures 7, 8 show no difference in average ligand association time and binding percentage with the two orientations. Because the binding pocket of HIVp is widely accessible for the ligand, the impact of the orientation was insignificant. However, some proteins exhibit a certain direction for ligands to enter the binding pocket, and their orientation against the flux of the ligands can affect the ligand–protein association.

### Artificially Localized HIVp 20 Å Above the Surface

Although different HIVp orientations did not affect xk263 association time, existing studies showed that the dimer interface (the bottom part of HIVp) is the most popular region for ligands' initial encounter of HIVp (Roberts and Chang, 2016). After reaching the dimer interface, the ligand can undergo surface diffusion to reach the binding pocket. When we placed HIVp 0 Å above the surface, this bottom region became partially inaccessible to xk263. Therefore, immobilized HIVp was artificially placed 20 Å above the surface so that xk263 can easily reach the bottom region. When the whole HIVp surface was accessible to xk263, the association time was reduced by 10–20% when the xk263 diffusion flux was <1.09 × 10^22^ molecules/s.m^2^, which suggests that the additionally available bottom region accelerates xk263 binding (Table 3) and slightly increases the association percentage (Table 4). Even when HIVp was placed 20 Å above the surface, the location was not far enough to eliminate the intermolecular xk263–SAM interactions. As a result, xk263 spent longer time near SAM when xk263 diffusion flux was small, thus resulting in longer time to associate with HIVp when SAM was present rather than absent. When the flux exists, the association time was the same when the SAM was present or absent, regardless the position of HIVp (Table 3).

## Conclusion

Ligand–protein encounters can occur in any environment that may provide additional intermolecular interactions or the transporting forces to the ligand. For example, in experimental settings such as using SPR to study ligand–protein binding, the choice of the surface and the flow rate are all optimized for measurements. In this study, we used BD simulations to investigate the effect of the CH_3_-SAM surface on xk263–HIVp association. The non-polar surface provided weak intermolecular vdW attractions with xk263 to slightly increase the ligand binding time. The effects quickly vanished when xk263 had an x-direction diffusion flux, and the association time decreased when the diffusion flux increased regardless of the presence of SAM or only a special plane. With no diffusion flux, xk263 was twice more concentrated within 20 Å of the SAM surface because of the xk263–SAM interactions. The concentration gradient did not increase binding time but increased xk263–HIVp binding probability. When the xk263 diffusion flux increased, the middle region of the square tube had the highest xk263 concentration, which is the same as existing experiments showing a quadratic curve of concentration gradients with flow. The results also show that when HIVp was placed on the surface (0 Å above the surface), the bottom part of the protein, a known high-probability site of the xk263–HIVp first encounter, was not accessible to xk263. Because xk263 could bind non-specifically on the HIVp surface and then utilize surface diffusion to reach the binding site, occluding this bottom region increased ligand binding time by 10–20% in the static environment. However, with large xk263 diffusion flux, all the weak intermolecular interactions and searching along the HIVp surface were eliminated, which resulted in a fast association time and small binding probability. This work brings insights into how ligand diffusion flux and the environment may affect ligand–protein association.

## Data Availability Statement

The raw data supporting the conclusions of this article will be made available by the authors, without undue reservation.

## Author Contributions

SK ran and analyzed the simulations, produced figures, and wrote the manuscript. C-eC designed experiments, analyzed the simulation, and wrote the manuscript. Both authors contributed to the article and approved the submission version.

## Conflict of Interest

The authors declare that the research was conducted in the absence of any commercial or financial relationships that could be construed as a potential conflict of interest.
