# Supplementary material for: Molecular Mechanics Study of Flow and Surface Influence in Ligand–Protein Association
Source: Front Mol Biosci. 2021 May 10;8:659687. doi: 10.3389/fmolb.2021.659687 (PMC8142692; doi:10.3389/fmolb.2021.659687)
Supplement: Supplementary file 1 [file Data_Sheet_1.PDF]

Supplementary Material

1 Supplementary Methods

1.1 Generating open conformation of HIVp

The structure of HIVp was obtained from Protein Data Bank (PDB) entry 1HHP. We used Amber 12 package for MD simulations of the protein with Amber 99SB and General Amber Force Field (GAFF). The charge of the system was neutralized by Cl^- counterions. The minimization of hydrogen atoms, the side chains, and the entire protein was performed before solvation. Then, the protein complex was solvated with a rectangular TIP3P water box with edge dimensions of 12 Å from the solutes. The system was slowly heated at 50, 100, 150, 200, 250, and 300K with an equilibrium course of 10 ps. We performed a 250ns simulation to obtain a variety of different conformations. The open conformation was appeared around 150 ns in the trajectory and was selected for this project.

1.2 Modeling surface assembled monolayer (SAM)

The SAM surface is consisted of undecanethiol chains on a gold sheet of 1 atom thickness. The size of the SAM was 400 x 400 $Å^2$ having a hexagonal packing pattern with a packing density in the order of 10^{14} cm^{-2}, which gives an average chain separation of 4.98 Å. The chains are attached to one-atom-thick Au(111) surface with Au-S bond. The structure of undecanethiol chain is corresponding to the lowest-energy conformation described by Schreiber(1). The chains are leaned 30° from the surface normal, 55 ^{o} twisted around their long axis, and 15 ^{o} rotated around the surface normal.

1.3 Comparison of modeled and analytical value of xk263's diffusion coefficient

Modeled Diffusion Coefficient- The model system used to calculate diffusion coefficient has periodic boundaries in both x- and y-direction and there is no termination at x-direction boundary. So, the trajectories used for calculations are continuous and 0.3μs long. We used the Einstein's relation, $<r^2>=2nDt$, to calculate the modeled diffusion calculation where r is the displacement in time t, n is the dimensionality and D is the diffusion coefficient.

$D_{modeled} = 5.58 \pm 0.33 \times 10^{-6}$ cm^2/s when a system had no SAM and no ligand diffusion flux.

Analytical Diffusion Coefficient- The analytical value for xk263 diffusion is based on Stokes-Einstein's relation, $D_{trans} = k_B T/6\pi\gamma r$. Here, we used radius of gyration as r to approximate the hydrodynamic radius.

where $k_B = 1.38\text{x}10^{-23}$ J/K, T=298K, γ=0.0008921 $Kgm^{-1}s^{-1}$, r = $4.98\text{x}10^{-10}$m

$D_{analytical} = 4.91\text{x}10^{6}$ cm^2/s.

1.4 Theoretical association time

We compared the simulation data to 3-dimention association rate theory. Theoretical 3D average association times, τ_3, depend highly on the radius of the final target a (HIVp's radius of gyration) and radius of the search space b (the simulation box or SAM) as well as the bulk or surface diffusion

coefficient D. With our simulation setting, a = 17.9 Å and b = 200 Å, we get 2648.4 ns association time.

$$\boldsymbol{\tau_3} = \left(\frac{\mathbf{b}^2}{\mathbf{D}}\right)\left(\frac{1 - \left(\frac{\mathbf{a}}{\mathbf{b}}\right)^2}{3\,\frac{\mathbf{a}}{\mathbf{b}}}\right)$$

1.5 Translational and rotational diffusion coefficients for overdamped langevin equation

To calculate the position after each timestep, translation and rotational diffusion coefficient (D_t and D_r) was computed using the Stokes-Einstein relation.

$$\mathbf{D_t} = \frac{\mathbf{k_B T}}{\mathbf{6\pi\gamma r}}$$

$$\mathbf{D_r} = \frac{\mathbf{k_B T}}{\mathbf{6\pi\gamma r^3}}$$

where k_B is Boltzmann's constant, T is the absolute temperature of the system, γ is the solvent viscosity, and r is the hydrodynamic radius (R_{hyd}).

2 Supplementary Figures

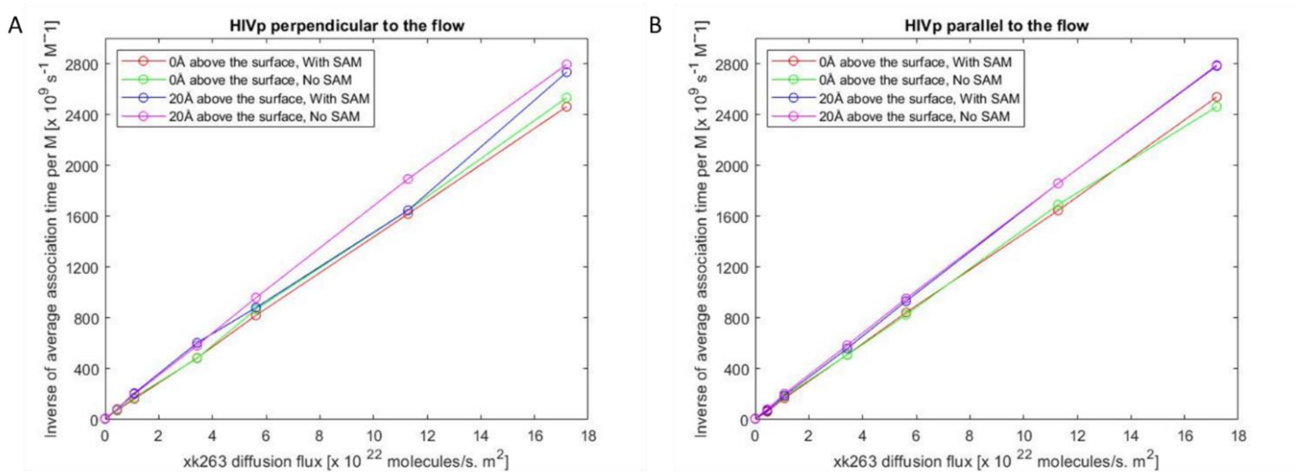

Supplementary Figure 1. Plot of inverse of average association time per molarity versus xk263 diffusion flux. Shows a linear relation of inverse of average association time per molarity with the diffusion flux.

3 Supplementary Tables

Value of N	xk263 diffusion flux (x 10^{22} /s.m^2)
0.00008	0.45±0.01
0.0002	1.09±0.07

0.0006	3.43±0.15
0.001	5.62±0.19
0.002	11.29±0.42
0.003	17.20±0.42

Supplementary Table 1. xk263 diffusion flux is the rate of molecules transferred across an imaginary plane per unit time per unit area. It was calculated using BD trajectory.

**xk263 diffusion flux (x 10^{22} /s.m^2)**	**With SAM, D (x 10^{-6} cm^2/s)**	**Without SAM, D (x 10^{-6} cm^2/s)**
**0**	4.71±0.44	5.58±0.33
**0.45**	4.52±0.22	5.50±0.28
**1.09**	4.64±0.36	5.70±0.34
**3.43**	5.60±0.22	6.62±0.29
**5.62**	7.60±0.29	8.92±0.39
**11.29**	17.49±0.71	19.08±0.45
**17.20**	33.89±0.82	35.51±1.17

Supplementary Table 2. Diffusion coefficient values of xk263 with and without CH_3-SAM under different diffusion flux.

References

1. F. Schreiber: Structure and growth of self-assembling monolayers. *Progress in surface science*, 65(5-8), 151-257 (2000)
